# The impact of combined gene mutations in *inhA* and *ahpC* genes on high levels of isoniazid resistance amongst *katG* non-315 in multidrug-resistant tuberculosis isolates from China

**DOI:** 10.1038/s41426-018-0184-0

**Published:** 2018-11-16

**Authors:** Liguo Liu, Fengting Jiang, Lihong Chen, Bing Zhao, Jie Dong, Lilian Sun, Yafang Zhu, Bo Liu, Yang Zhou, Jian Yang, Yanlin Zhao, Qi Jin, Xiaobing Zhang

**Affiliations:** 1 MOH Key Laboratory of Systems Biology of Pathogens, Institute of Pathogen Biology, Chinese Academy of Medical Sciences & Peking Union Medical College, 100176 Beijing, China; 2 Chinese Center for Disease Control and Prevention, 155# Changbai Road, Changping District, 102206 Beijing, China

## Abstract

Whole-genome sequencing was used to analyze the profiles of isoniazid (INH) resistance-related mutations among 188 multidrug-resistant strains of *Mycobacterium tuberculosis* (MDR-TB) and mono-INH-resistant isolates collected in a recent Chinese national survey. Mutations were detected in 18 structural genes and two promoter regions in 96.8% of 188 resistant isolates. There were high mutation frequencies in *katG*, the *inhA* promoter, and *ahpC-oxyR* regulator regions in INH-resistant isolates with frequencies of 86.2%, 19.6%, and 18.6%, respectively. Moreover, a high diversity of mutations was identified as 102 mutants contained various types of single or combined gene mutations in the INH-resistant group of isolates. The cumulative frequencies of *katG* 315 or *inhA*-P/*inhA* mutations was 68.1% (128/188) for the INH-resistant isolates. Of these isolates, 46 isolates (24.5% of 188) exhibited a high level of resistance. A high level of resistance was also observed in 21 isolates (11.2% of 188) with single *ahpC-oxyR* mutations or a combination of *ahpC-oxyR* and *katG* non-315 mutations. The remaining 17 mutations occurred sporadically and emerged in isolates with combined *katG* mutations. Such development of INH resistance is likely due to an accumulation of mutations under the pressure of drug selection. Thus, these findings provided insights on the levels of INH resistance and its correlation with the combinatorial mutation effect resulting from less frequent genes (*inhA* and/or *ahpC*). Such knowledge of other genes (apart from *katG*) in high-level resistance will aid in developing better strategies for the diagnosis and management of TB.

## Introduction

Tuberculosis (TB) is caused by infection with *Mycobacterium tuberculosis* and represents one of the greatest threats to human health worldwide, and it is associated with 1.4 million deaths annually (2016 WHO report). As a component of first-line TB drugs, isoniazid (INH) is used both for the treatment of active TB and as a preventive therapy for latent infections^1^. The emergence of INH-resistant *M. tuberculosis* strains readily leads to the development of multidrug-resistant TB (MDR-TB; *M. tuberculosis* strains resistant to INH and rifampicin)^2^. Thus, the transmission of MDR-TB presents a challenge for both TB treatment and prevention globally. A rapid molecular diagnosis is an important strategy for preventing both the transmission of resistant strains and further development of drug resistance during TB treatment. However, a clear understanding of the underlying mechanisms of resistance and the identification of new markers for the diagnosis of drug-resistant TB are required.

INH is a prodrug activated by catalase-peroxidase, which is encoded by *katG*^3^. The molecular basis of INH resistance in *M. tuberculosis* was first revealed by the identification of *katG* deletion in clinical INH-resistant isolates^4^. INH resistance also involves several genes in multiple biosynthetic networks and pathways^5^. Activated INH functions by attacking a series of components involved in the biosynthesis of mycolic acids, such as NADH-dependent enoyl ACP synthase (encoded by *inhA*), β-ketoacyl ACP synthase (encoded by *kasA*), malonyl-CoA acyl carrier protein (ACP) transacylase (*fabD*), and acetyl-CoA carboxylase (*accD6*)^6,7^. Thus, *M. tuberculosis* strains with mutations in these genes may develop INH resistance^5^. In addition, 15 other structural genes and regulator regions have also been found to be involved in INH resistance^8^. For example, mutations in peroxiredoxin alkyl hydroperoxide reductase subunit C (*ahpC)* and the *ahpC-oxyR* intergenic regulatory region have been identified in INH-resistant isolates^6^. Moreover, the ferric uptake regulator gene (*furA*) and several efflux protein-encoding genes (*iniABC* and *efpA*) have been shown to be mutated in INH-resistant *M. tuberculosis* isolates^9–12^. Investigators have also studied other efflux (mmpL family) genes and their significance in INH resistance^13^. Additionally, mutations in trehalose dimycolyl transferase (*fbpC*), a regulatory gene governing the expression of a polyketide synthase (*srmR*), as well as in *Rv0340*, *Rv1592c, Rv1772*, *fadE24*, *ndh*, and *nat* have been identified in resistant *M. tuberculosis* strains^14–17^.

Although a variety of genes is involved in *M. tuberculosis* resistance to INH, there is data to support that frequent mutations are primarily focused in the *katG*, *inhA*, and *ahpC-oxyR* regulator regions^18^. Information regarding other gene mutations is limited because it is challenging to simultaneously acquire all gene sequences related to INH resistance in a collection of isolates, which creates a gap in the understanding of the accumulation of several gene mutations in the development of INH-resistance during TB treatment. Whole genome sequencing (WGS) was used to analyze the mutation profile of 22 known genes in clinical *M. tuberculosis* isolates. In the present study, 188 MDR isolates and mono-INH-resistant isolates were selected from a collection generated in a national survey^19^. The occurrence of MDR-TB strains is largely associated with the development of INH mono-resistance under the continuous pressure of drug selection during TB treatment^20^. The high degree of polymorphisms among these mutations provides an opportunity to study the relationship between mutations and the level of phenotypic resistance. The aim of the present study was to explore the profile of gene mutations related to INH resistance in MDR-TB isolates. Comparing the degree of phenotypic drug resistance among these isolates revealed the influence of accumulated gene mutations in *M. tuberculosis* strains during the development of INH resistance in patients with MDR-TB. Identification of high levels of mutations associated with resistance helps to establish new reliable molecular methods for the detection of INH drug resistance.

## Results

Scanning the whole-genome sequence resulted in detection of drug resistance-related mutations in 18 structural genes and two promoter regions in 96.8% (182/188) of the INH-resistant isolates (Table 1). Mutations in *katG* were detected in 86.2% (162/188) of the INH-resistant isolates. Mutations in the *inhA/inhA* promoter (*inhA*-P) and *ahpC-oxyR* regulator region occurred in 19.6% (37/188) and 18.6% (35/188) of isolates, respectively. These three gene loci were regarded as highly frequent gene loci. The occurrence of the remaining genes ranged from 0.53% to 4.26% of isolates and were classified as low frequent genes.

**Table 1:** Mutation frequencies for multiple INH resistance-related genes and regulator regions

Gene locus	Functional description	Frequency (*n* = 188) (%)	MDR^a^	mono-R^b^	Types of mutations	Substitution	Deletions	SNP in susceptible^c^
*katG* (Rv1908c)	Catalase-peroxidase-peroxynitritase	86.17	144	18	59	57	2	R463L
*inhA*-promoter	Promoter region	19.15	34	2	6	6	0	—
*ahpC-oxyR* (Rv2427-2428)	Regulator region	18.62	33	2	11	11	0	—
*inhA* (Rv1484)	NADH-dependent enoyl-ACP reductase	2.66	5	0	2	2	0	—
*aphC* (Rv2428)	Alkyl hydroperoxidase C	0.53	1	0	1	1	0	—
*iniA* (Rv0342)	INH-inducible gene	4.26	6	2	5	5	0	G178(.)
Rv1592c	Unknown	4.26	7	1	5	5	0	I322V + E321(.)
*furA* (Rv1909c)	Ferric uptake regulator	3.72	5	2	5	5	0	—
*fabD* (Rv2243)	Malonyl-CoA ACP transacylase	2.66	5	0	2	2	0	P179A
Rv1772	Unknown	2.66	5	0	1	1	0	—
*nhoA/nat* (Rv3566c)	Arylamine *N*-acetyltransferase	2.66	4	1	1	1	0	G207R
*efpA* (Rv2846c)	Efflux protein	2.13	4	0	4	4	0	—
*kasA* (Rv2245)	β-Ketoacyl ACP synthase	2.13	4	0	2	2	0	H253Y
*iniC* (Rv0343)	INH-inducible gene	1.60	3	0	3	3	0	A21(.); P22(.)
*fadE24* (Rv3139)	Fatty acyl-CoA dehydrogenase	1.06	2	0	2	2	0	—
*srmR* (Rv2242)	Regulatory gene	1.06	2	0	2	2	0	M323T
*fbpC* (Rv0129c)	Trehalose dimycolyl transferase	1.06	2	0	2	2	0	G158S;P237S
*iniB* (Rv0341)	INH-inducible gene	0.53	1	0	1	1	0	—
*ndh* (Rv1854c)	NADH dehydrogenase	0.53	1	0	1	1	0	—
Rv0340	Unknown	0.53	1	0	1	1	0	—
*accD6* (Rv2247)	Acetyl-CoA carboxylase	0.00	0	0	—			—
*fabG1* (Rv1483)	3-ketoacyl-acyl carrier protein reductase	0.00	0	0	—			—

^a^Number of MDR isolates

^b^Number of INH mono-resistant isolates

^c^These types of mutations occurred simultaneously in MDR isolates and susceptible isolates, which were not included in the frequency of INH-resistant mutations

A high degree of polymorphism in mutation types was displayed in the collection. There were 102 mutant types, including 28 single-gene mutations and 74 combined gene mutations involved in the 18 INH resistance-related gene loci (Table 2). Single-gene mutations were identified in 83 (49.4% of 168) MDR-TB isolates and 14 (70% of 20) mono-resistant isolates. Two, three, four, and five gene mutations were in 31.9% (60/188), 9.0% (17/188), 2.7% (5/188), and 0.5% (1/188) of the INH-resistant isolates, respectively. Moreover, there were six isolates for which INH resistance-related mutations were not detected.

**Table 2:** Occurrence of various combined mutations determined in isolates with different resistance levels

Combined gene mutations (Gene loci)^a^					Num. of isolates (%)	Num. of mutant types	Type of isolates	LR^b^	MR^b^	HR^b^
1st	2nd	3rd	4th	5th				(0.1 mg/L < MICs ≤ 0.4 mg/L)	(0.8 mg/L ≤ MICs ≤ 3.2 mg/L)	MICs ≥ 6.4 mg/L)
*katG315*					66(35.11)	5	MDR, mono-R	1	42	23
*katG315*	+*inhA-P*				6(3.19)	4	MDR	0	0	6
*katG315*	*+iniA*				6(3.19)	3	MDR, mono-R	0	2	4
*katG315*	*+* *kasA*				3(1.60)	3	MDR	0	1	2
*katG315*	*+* *Rv1592c*				3(1.60)	3	mono-R	0	2	1
*katG315*	*+ahpC-oxyR*				2(1.06)	2	mono-R	0	1	1
*katG315*	*+fabD*				2(1.06)	2	MDR	0	1	1
*katG315*	*+furA*				1(0.53)	1	MDR	0	1	0
*katG315*	*+Rv1772*				1(0.53)	1	MDR	0	0	1
*katG315*	*+ndh*				1(0.53)	1	MDR	0	1	0
*katG315*	*+inhA-P*	*+Rv1772*			1(0.53)	1	MDR	0	0	1
*katG315*	*+fabD*	*+iniC*			1(0.53)	1	MDR	0	1	0
*katG315*	*+furA*	*+Rv1592*			1(0.53)	1	MDR	0	1	0
*katG315*	*+iniA*	*+nat*			1(0.53)	1	mono-R	0	1	0
*katG315*	*+iniC*	*+efpA*	*+Rv0340*		1(0.53)	1	MDR	0	1	0
*katG315*	*+ahpC-oxyR*	*+fbpC*	*+fadE24*	*+srmR*	1(0.53)	1	MDR	0	1	0
*katGnon315*					16(8.51)	16	MDR, mono-R	5	9	2
*katGnon315*	*+inhA-P*				13(6.91)	8	MDR	3	8	2
*katGnon315*	*+ahpC-oxyR*				13(6.91)	13	MDR	2	7	4
*katGnon315*	*+Rv1592c*				2(1.06)	2	MDR	1	0	1
*katGnon315*	*+inhA*				1(0.53)	1	MDR	1	0	0
*katGnon315*	*+fabD*				1(0.53)	1	MDR	0	1	0
*katGnon315*	*+furA*				1(0.53)	1	mono-R	0	1	0
*katGnon315*	*+Rv1772*				1(0.53)	1	MDR	0	1	0
*katGnon315*	*+efpA*				1(0.53)	1	MDR	0	1	0
*katGnon315*	*+nat*				1(0.53)	1	MDR	1	0	0
*katGnon315*	*+inhA-P*	*+inhA*			3(1.60)	2	MDR	0	3	0
*katGnon315*	*+inhA-P*	*+Rv1592c*			1(0.53)	1	MDR	0	1	0
*katGnon315*	*+inhA-P*	*+Rv1772*			1(0.53)	1	MDR	0	0	1
*katGnon315*	*+inhA-P*	*+ahpC-oxyR*			1(0.53)	1	MDR	0	1	0
*katGnon315*	*+inhA-P*	*+ahpC-oxyR*	*+furA*		2(1.06)	2	MDR, mono-R	0	2	0
*katGnon315*	*+ahpC-oxyR*	*+ahpC*			1(0.53)	1	MDR	0	0	1
*katGnon315*	*+ahpC-oxyR*	*+furA*			1(0.53)	1	MDR	0	1	0
*katGnon315*	*+ahpC-oxyR*	*+iniA*			1(0.53)	1	MDR	0	0	1
*katGnon315*	*+ahpC-oxyR*	*+kasA*			1(0.53)	1	MDR	0	0	1
*katGnon315*	*+ahpC-oxyR*	*+nat*			1(0.53)	1	MDR	0	1	0
*katGnon315*	*+ahpC-oxyR*	*+Rv1592c*			1(0.53)	1	MDR	0	0	1
*katGnon315*	*+ahpC-oxyR*	*+efpA*	*+fadE24*		1(0.53)	1	MDR	0	1	0
*inhA-P*					7(3.72)	1	MDR, mono-R	3	3	1
*inhA-P*	*+Rv1772*	*+furA*			1(0.53)	1	MDR	0	0	1
*inhA-P*	*+inhA*	*+ahpC-oxyR*	*+ahpC*		1(0.53)	1	MDR	1	0	0
*ahpC-oxyR*					5(2.66)	4	MDR	0	0	5
*ahpC-oxyR*	*+iniB*				1(0.53)	1	MDR	0	0	1
*ahpC-oxyR*	*+iniC*	*+efpA*			1(0.53)	1	MDR	1	0	0
*ahpC-oxyR*	*+fbpD*	*+fabD*			1(0.53)	1	MDR	0	0	1
*nat*					2(1.06)	1	MDR, mono-R	1	0	1
*srmR*					1(0.53)	1	MDR	0	0	1
Wild types					6(3.19)	0	MDR	0	4	2
Total					188	102	—	20	101	67

^a^Combination of multiple gene mutations present in isolates: “+” represents the simultaneous existence of mutations in each isolate

^b^LR, MR, and HR refer to low-level INH resistance, intermediate-level INH resistance, and high-level INH resistance, respectively

### Single gene mutations

In the collection, single gene mutations were detected in five gene loci from 97 isolates. The most frequent mutations occurred in *katG*, and they were detected in 84.5% (82/97) of the isolates. Three types of substitutions involving amino acids at position 315 were detected in 66 (68% of 82) isolates, whereas two types of double substitutions occurred in *katG*. Ser315Thr was prevalent and was identified in 56 (84.8% of 66) isolates. Ser315Asn and Ser315Arg were found in nine isolates (13.6%) and one isolate (1.5%), respectively. Single Ser315Thr and Ser315Asn were observed in the median of MICs at levels of 3.2 and 12.8 mg/L, respectively.

**Fig. 1:**
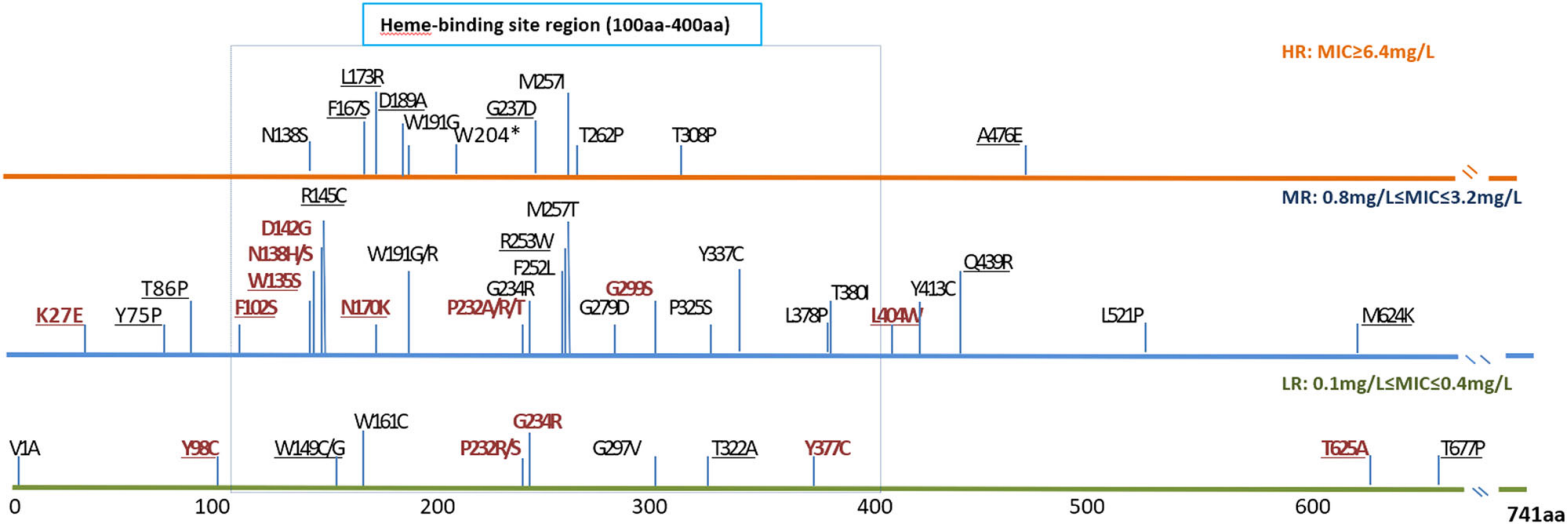
Distribution of non-315 mutations (substitutions) in katG. These mutations were divided into three groups based on the level of phenotypic INH resistance displayed by the isolates. The different colored lines indicate the corresponding resistance level as follows: mutations conferring low-level resistance (LR) are shown in green; mutations conferring intermediate-level resistance (MR) are shown in blue; and mutations conferring high-level resistance (HR) are shown in orange. Single gene mutations are shown in red, and combined gene mutations are shown in black. To the best of our knowledge, underlined mutations have not been previously reported

Single *katG* mutations at codons other than codon 315 (termed non-315 mutations) were detected in 16 isolates, which were represented by 16 mutant types in 13 amino acid positions (Fig. 1). Except for three types, all mutations occurred in the N-terminus of the KatG protein from amino acid position 27 to 299. Two types of substitutions occurred at position 138 as follows: Asp138His and Asp138Ser. At position 232, four types of substitutions (Pro232Ala/Arg/Ser/Thr) were detected in the isolates. The MIC median was 1.2 mg/L in the isolates with non-315 *katG* mutations. Nearly all intermediate-level resistance was related to mutations that occurred in positions prior to amino acid 299.

Single gene mutations were also detected in two regulator regions, namely *inhA-*P and *ahpC-oxyR*. In the *inhA-P* mutations, -15C-T was the predominant mutant type and was detected in all seven isolates. The median MIC value was 0.8 mg/L for -15C-T mutations. In all except one (MICs = 25.6 mg/L) of the remaining isolates, the MIC was below 1.6 mg/L. *ahpC-oxyR* mutations presented as four types of nucleotide substitutions (-48G-A, -51G-A, -57C-T, and -81C-T) in five isolates. The median MIC was 51.2 mg/L, and all MICs were above 12.8 mg/L in these isolates.

In addition, mutations in *nat* and *srmR* were represented by the substitution of Gly207Arg and A224V, which were separately detected in two and one isolates, respectively. A high variation of MIC results was observed in these three isolates, ranging from 0.1 to 102.4 mg/L.

### Combined gene mutations

Combined gene mutations were identified in 79 (47% of 168) MDR-TB isolates and six (30% of 20) isolates with INH mono-resistance. In all but five isolates, the mutations involved a combination of *katG* mutations with the other 16 gene loci (Table 2).

**Fig. 2:**
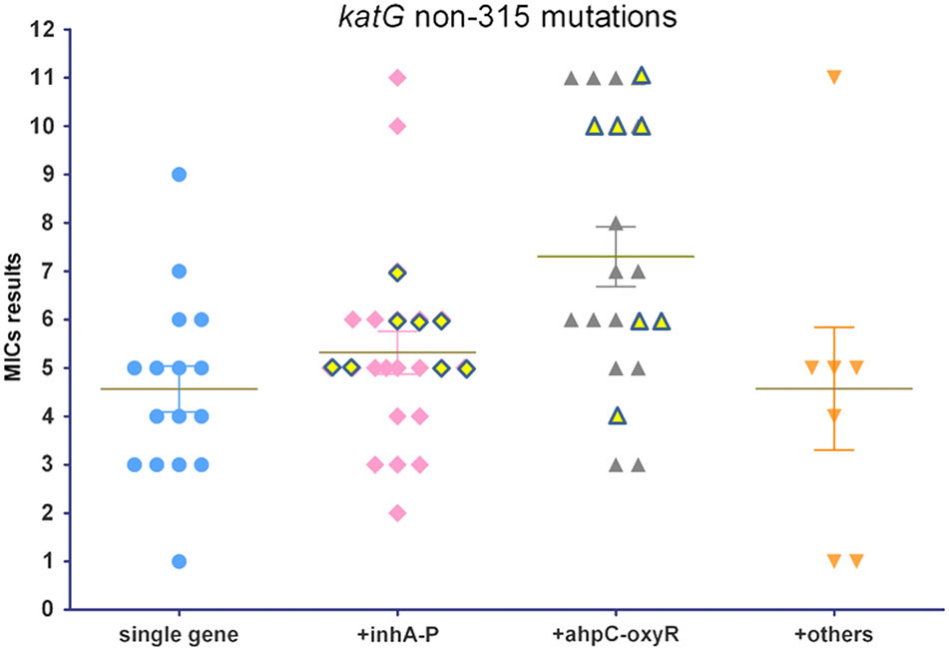
MIC results for INH-resistant isolates with katG non-315 mutations. The horizontal axis shows four groups of isolates with single gene mutations and different combinations of multiple gene mutations. MIC results are represented by numbers from 1 to 11 on the vertical axis, corresponding to 11 double diluted concentrations of INH, ranging from 0.1 to 102.4 mg/L. The yellow diamond and triangle shapes with outskirts refer to the MIC results from isolates with three or four simultaneous gene mutations involved in the less frequent gene loci

Combined 315 mutations were detected in 32% (31/97) of the isolates. Mutations involving two genes were the predominant form of combination and were identified in 80.6% (25/31) of the isolates. Mutations involving more than three gene mutations were found in the remaining six isolates. Simultaneous mutations in *inhA-*P were the most frequent and were detected in seven (22.6% of 31) isolates. All of these isolates exhibited a high level of resistance with MIC results above 6.4 mg/L. Among these isolates, nucleotide substitutions of -15C-T, -8T-A(C), and -34C-T were detected. These isolates also displayed greater resistance compared to the other combined gene mutations (Fig. 2a).

It should be noted that mutations in efflux pump genes were simultaneously detected from the combined *katG* mutations. There were *iniA* mutations in seven isolates, and there were *iniC* mutations in two isolates. Four isolates with *katG* 315 mutations combined with *iniA* Gln26Arg and Arg603Lue mutations showed a high level of resistance. In addition, only 40% (6/15) of isolates displayed a high level of resistance among these combined mutations with the remaining genes.

Moreover, combined *katG* non-315 mutations were detected in 75.4% (49/65) of the isolates. Simultaneous mutations occurred in 12 other genes. A high frequency of mutations was detected in *inhA*-P and/or *ahpC-oxyR* in 87.8% (42/49) of the combined non-315 mutations (Fig. 2b). A combination of non-315 and *inhA*-P mutations was observed in 13 isolates with two gene mutations, six isolates with three gene mutations, and two isolates with four gene mutations. A combination of *katG* non-315 with *ahpC-oxyR* mutations was found in 13 isolates with two gene mutations, six isolates with three gene mutations, and three isolates with four gene mutations.

Among these combined *katG* non-315 mutations, the highest MIC median was detected in isolates with a simultaneous occurrence of *ahpC-oxyR* mutations. The MIC median was as high as 3.2 mg/L, which was higher than other types of the combined *inhA*-P mutations (Fig. 2) that also occurred at a high frequency. Moreover, of these combined *katG* non-315 mutations, the frequency of HR isolates was greater than among those with combined *inhA*-P mutations (39.1% [9/23] and 19.0% [4/21], respectively). In addition to mutations in these two regulator regions, simultaneous mutations in 10 other structural genes were present in the non-315 mutations.

Apart from these combined *katG* mutations, there were five isolates with combined gene mutations in *ahpC-oxyR* or *inhA*-P that simultaneously occurred with other genes (Table 2). Three isolates involved the combination of mutations in *ahpC-oxyR* with one or two other genes, such as *iniB, fabD* and *fbpC, iniC*, and *efpA*. One isolate exhibited a combination of mutations in *inhA*-P with *furA* and *Rv1772*. A combined substitution of the regulator region and structural gene of *inhA*-P/*inhA* and *ahpC-oxyR*/*ahpC* was observed, which was -15C-T/S94A/-11T-C/E189D.

### Mutations simultaneously detected from pan-susceptible isolates and INH-resistant isolates

In addition to the mutations observed in the INH-resistant isolates, eight types of amino acid substitutions were simultaneously identified in both the susceptible and resistant isolates. There were seven types of substitutions detected in the collection as follows: Arg463Leu in *katG*; Ile322Val in *Rv1592*; Pro179Ala in *fabD*; Gly207Arg in *nat*; His253Tyr in *kasA*; Gly158Ser and Pro237Ser in *fbpC*; and Met323Thr in *srmR*.

Based on a phylogenetic analysis, three lineages of INH-resistant isolates were detected. Aside from one strain belonging to lineage 3, 145 isolates were of lineage 2, and 22 isolates were classified into lineage 4. The isolates belonging to lineage 2 contained the *katG* Arg463Leu substitution, which is the standard genetic marker for identifying Beijing family strains.

## Discussion

The mechanism of *M. tuberculosis*-mediated INH resistance is highly complex due to the involvement of several genes. The present study focused on illustrating the relationship between various kinds of combined mutations and the associated phenotypic resistance. Using a WGS platform, mutations in these genes were simultaneously detected for each isolate in the collection. These results provided an opportunity to discover the influence of these gene mutations on the development of INH resistance in MDR-TB isolates.

Previous published data have demonstrated that *katG* 315 mutations, especially Ser315Thr, are the main cause of INH resistance, exhibiting a frequency ranging from 42% to over 90% in different regions throughout the world^18^. Currently, *katG* 315 mutations and mutations in *inhA* structural and promoter regions are used as markers for the rapid detection of INH resistance. Commercial kits (e.g., Geno Type MTBDR Plus Assay and the Nipro NTM + MDRTB Detection Kit 2) have been recommended by the World Health Organization for the diagnosis of INH resistance in clinical isolates (WHO, 2016). In the present collection, there was a predominance of *katG* mutations, which were detected in 86.2% of the INH-resistant isolates, far higher than that of other genes. In comparison, a substitution of Ser315Thr was only identified in 41.0% (69/168) of the MDR-TB isolates and 65% (13/20) of the INH mono-resistant isolates. This finding indicated that the more complex INH-resistance mutations occurred in MDR-TB isolates. Indeed, a high diversity of *katG* mutations was exhibited by both types of deletions and 53 amino acid substitutions. These mutations were represented as 90 types of mutations, including 21 different single gene mutations and 69 combined gene mutations (Table 2). Except for two genes, nearly all INH resistance-related genes occurred in combination with *katG* mutations.

The prevalence of 315 mutants may be due to the loss of oxidase activities but retainment of the catalase-peroxidase activities of *katG*. Moreover, because this modification confers a survival advantage, it is readily spread throughout the population^21,22^. The present study showed that isolates with single 315 mutations displayed intermediate levels of resistance (median MIC = 3.2 mg/L). In addition, isolates with combined 315 mutations exhibited a higher MIC median, and HR isolates occurred with a higher frequency than those with single 315 mutations.

The accumulation of other gene mutations increased the resistance to INH among these MDR-TB isolates. It should be noted that *inhA-*P and efflux pump encoding gene (*iniA*) mutations were responsible for this development of drug resistance. Within these MDR-TB strains, INH resistance has been found to be associated with the overexpression of *InhA*, which is caused by mutant *inhA*-P^23^. As a putative target for INH and ethionamide, overexpression of the NADH-specific enoyl-ACP reductase may result in more active INH^24^. A previous study has also shown a selective advantage for strains harboring *inhA*-P mutations to become XDR-TB strains using the current treatment regimen^25^. Moreover, Ser315Thr has also been reported to be associated with an unfavorable treatment outcome, and *inhA-P* increases the risk of relapse^26^. Thus, the detection of this type of mutation combination in MDR-TB isolates indicates how high-level resistance emerges and supports the more cautious usage of anti-TB drugs for treatment.

Recent studies have also shown that *katG* non-315 substitutions are a frequent occurrence among INH-resistant isolates^27,28^. In the present collection, several non-315 mutations were detected among the MDR-TB isolates. Of these mutations, 23 have not been previously reported (Fig. 1). Moreover, these non-315 mutations occurred throughout the entire *katG* gene. The non-315 mutations located in the N-terminus of the *KatG* protein typically exhibited intermediate or high resistance. These findings suggested that this region is critical for INH activity, and various degrees of reduction in catalase activity are also associated with INH resistance^29^.

Compared to the majority of single genes involved in *katG* 315 mutations, non-315 mutations commonly occurred with further accumulation of other gene mutations. More than two-thirds of isolates with non-315 mutations simultaneously harbored other gene mutations. Two gene loci consisted of *inhA*-P and *ahpC-oxyR*, which were frequently identified in these isolates. Using line probe assays, the positive detection of only *inhA*-P mutations represented low-level resistance. These results also demonstrated that single *inhA-P* mutations were related to low-level resistance. However, the frequency of intermediate-level resistance increased substantially in isolates with non-315 mutations combined with *inhA-P* mutations (from 42.9% [3/7] to 68.4% [13/19]).

A previous study has demonstrated that mutations in *ahpC-oxyR* are compensatory alterations that occur due to a loss in catalase-peroxidase activity^30^. Mutations in *ahpC-oxyR* occur at a low frequency due to limited data obtained from previous detections^18^. In the present study, a substantial number of MDR-TB isolates harbored mutations in this regulator region, and the majority of which displayed a high level of resistance with MIC results over 6.4 mg/L. Among these isolates, *ahpC-oxyR* mutations were located in the region from −48 to −54. In contrast, combined non-315 mutations were located in the heme-binding site region, which is the active site structure for catalase-peroxidase function^31^. If there was a compensatory effect produced by *ahpC-oxyR* mutations, a high level of resistance would be associated with these non-315 amino acid substitutions. These findings revealed that these amino acids are likely key positions in *katG* because these mutations are associated with a greater loss of KatG function and require overexpression of *ahpC* for mycobacteria to resist the pressure of high INH concentrations. In addition, the occurrence of *ahpC-oxyR* is associated with relapse or treatment failure. Moreover, a higher frequency of *ahpC-oxyR* mutations was present in isolates from retreated MDR-TB patients compared to isolates from new cases (33.0% [34/103] vs. 17.6% [15/85]; *P* *<* 0.05). One notable phenomenon in the present study was that *inhA*-P or *ahpC-oxyR* mutations occurred in all non-315 mutations combined with three or four gene mutations, and these isolates typically displayed relatively high MICs (Fig. 2). This finding indicated that these less frequent gene mutations occur as a subsequent accumulation during the development of INH resistance among these isolates.

Genotyping demonstrated that *katG* 315 mutations frequently occurred in isolates belonging to lineage 2, also known as the Beijing family. Moreover, the majority of combined 315 mutations belonged to sublineage 2.3, which are prevalent strains in China^32^. However, *katG* non-315 mutations were also found in isolates belonging to other lineages, such as Lineage 3 and 4. The high degree of polymorphisms among non-315 mutations reflected the development of drug resistance resulting from the accumulation of mutations in multiple gene loci under a selection pressure. This process may be strongly associated with the failure or relapse of TB treatment.

The present study determined the mutation profile of INH resistance-related gene mutations in MDR-TB and mono-resistant isolates. Although several gene loci are involved in INH resistance, isolates exhibiting high-level resistance were typically associated with the accumulation of *katG* mutations combined with *inhA-P* and *ahpC-oxyR* mutations. In addition to 315 mutations, combined *katG* non-315 and *ahpC-oxyR* mutations also revealed a close relationship with a high level of resistance in MDR-TB isolates. However, this group of mutations could not be detected with a commercial kit for the diagnosis of INH resistance. Such mutations have not been of great concern in previous studies due to limited data regarding the occurrence in clinical isolates. Moreover, the remaining gene mutations occurred occasionally and usually as a component of combined mutations in MDR-TB isolates. These findings expanded the understanding of the development of INH resistance by *M. tuberculosis* in TB patients.

## Materials and methods

### Clinical isolates

To acquire a high diversity of mutant types with gene mutations associated with INH resistance, *M. tuberculosis* isolates from a national survey of drug resistance recently conducted in China were selected for analysis. A collection of 201 isolates was randomly obtained from the Chinese national survey of the prevalence of drug-resistant TB conducted in 2007, which included 168 MDR-TB isolates, 20 INH mono-resistant isolates, and 13 pan-susceptible isolates. These isolates were obtained from TB patients in 72 representative regions. Thus, high polymorphism of INH resistance-related mutations was represented in these isolates.

Single clones for each isolate were cultured on Löwenstein–Jensen medium^33^. Original cultures were collected, and 10-fold serial dilutions were prepared. These dilutions were then cultured and harvested to extract genomic DNA. The present study was approved by the Ethics Review Committee of the Institute of Pathogen Biology, Chinese Academy of Medical Sciences & Peking Union Medical College.

### Drug susceptibility testing and determination of MDR and INH mono-resistant strains

The drug resistance profile of each isolate was determined using the absolute concentration method on Löwenstein–Jensen medium^19^. Six key anti-tuberculosis drugs (isoniazid, 0.2 mg/L; streptomycin, 4 mg/L; rifampicin, 40 mg/L; ethambutol, 2 mg/L; ofloxacin, 2 mg/L; and kanamycin, 40 mg/L) from Sigma Aldrich were selected for drug susceptibility testing using concentrations based on the WHO guidelines. MDR strains were those determined to exhibit resistance to both INH and rifampicin. Mono-INH-resistant isolates were defined as those resistant only to INH and susceptible to the other five drugs. Pan-susceptible isolates were susceptible to all six drugs. Further phenotypic confirmation of each type of isolate was performed using the BACTEC MGIT 960 System (BD Diagnostic Systems).

Determination of minimum inhibitory concentration (MIC) was performed in 96-well microplates using the colorimetric method^34^. Isolates were treated with 10 INH concentrations, ranging from 0.05 to 102.4 mg/L, which were prepared by two-fold dilution in 7H9 broth. MIC results were read as the lowest concentration of INH that prevented a color change for each isolate.

The isolates were divided into four groups according to the degree of INH resistance as follows: 1) susceptible (MIC < 0.01 mg/L); 2) low-level resistance (LR; 0.1 mg/L ≤ MIC ≤ 0.4 mg/L); 3) intermediate-level resistance (MR; 0.8 mg/L ≤ MIC ≤ 3.2 mg/L); and 4) high-level resistance (HR; MIC ≥ 6.4 mg/L).

### Identification of INH resistance-related mutations using next-generation sequencing

Genomic DNA from each isolate was extracted using a Wizard Genomic DNA Purification Kit (Promega, Co., Madison, USA). Sequencing libraries were constructed and sequenced with a Truseq ® Nano DNA kit (Illumina, Inc., San Diego, CA, USA). Quality assessment of the sequencing data was performed using the NGS QC Toolkit with a cutoff of Q20, and a minimum read length of 101 base pairs was used for subsequent mapping. Valid reads were mapped to the reference genome sequence of *M. tuberculosis* H37Rv (GenBank accession NC_000962) using the Burrows–Wheeler algorithm as implemented in the BWA software package^35^. For all isolates, the reference genome coverage was >99% with a minimum depth of 10× and a consensus quality score of 50 using SAMtools. Mutations in each INH resistance-related gene were identified by aligning the corresponding reads with the reference sequence (*M. tuberculosis* H37Rv). Sequencing reads have been submitted to the NCBI sequence read archive (SRA) under accession PRJNA268900.

### Statistical analysis

The Chi-squared test or Mann–Whitney *U*-test was used when appropriate to assess the relationship between the mutations and MICs. Significance was considered when *P* < 0.05. SPSS statistical software package was used for all statistical analyses.
